# Hyper-inflammatory profile and immunoparalysis in patients with severe Legionnaires’ disease

**DOI:** 10.3389/fcimb.2023.1252515

**Published:** 2023-10-27

**Authors:** Camille Allam, William Mouton, Hugo Testaert, Christophe Ginevra, Noémie Fessy, Marine Ibranosyan, Ghislaine Descours, Laetitia Beraud, Johann Guillemot, Annelise Chapalain, Chloé Albert-Vega, Jean-Christophe Richard, Laurent Argaud, Arnaud Friggeri, Vanessa Labeye, Yvan Jamilloux, Nathalie Freymond, Fabienne Venet, Gérard Lina, Patricia Doublet, Florence Ader, Sophie Trouillet-Assant, Sophie Jarraud

**Affiliations:** ^1^ Centre National de Référence des Légionelles, Hôpital de la Croix-Rousse, Hospices Civils de Lyon, Lyon, France; ^2^ Centre International de Recherche en Infectiologie (CIRI), Legiopath team, Inserm, U1111, CNRS, UMR5308, Ecole Normale Supérieure de Lyon, Université Claude Bernard Lyon 1, Lyon, France; ^3^ Unité Mixte de Recherche Hospices Civils de Lyon-bioMérieux, Pierre-Bénite, France; ^4^ Centre International de Recherche en Infectiologie (CIRI), Virpath Team Inserm U1111, CNRS UMR5308, ENS Lyon, Université Claude Bernard Lyon 1, Lyon, France; ^5^ Service de Médecine Intensive-Réanimation - Hôpital de la Croix-Rousse, Hospices Civils de Lyon, Lyon, France; ^6^ Service de Médecine Intensive-Réanimation - Hôpital Edouard Herriot, Hospices Civils de Lyon, Lyon, France; ^7^ Département d’Anesthésie Réanimation - Centre Hospitalier Lyon Sud, Hospices Civils de Lyon, Pierre-Bénite, France; ^8^ Service des urgences - Hôpital de la Croix-Rousse, Hospices Civils de Lyon, Lyon, France; ^9^ Département de Médecine Interne, Hôpital de la Croix-Rousse, Hospices Civils de Lyon, Lyon, France; ^10^ Service de Pneumologie, Centre Hospitalier Lyon Sud - Hospices Civils de Lyon, Pierre-Bénite, France; ^11^ Laboratoire d’Immunologie - Hôpital Edouard Herriot - Hospices Civils de Lyon, Lyon, France; ^12^ Centre International de Recherche en Infectiologie (CIRI), NLRP3 Inflammation and Immune Response to Sepsis, Inserm U1111, CNRS, UMR5308, Ecole Normale Supérieure de Lyon, Université Claude Bernard-Lyon 1, Lyon, France; ^13^ Département des Maladies Infectieuses et Tropicales - Hôpital de la Croix-Rousse, Hospices Civils de Lyon, Lyon, France

**Keywords:** Legionnaires’ disease, hyper-inflammation, immunoparalysis, immune functional assays, severity, LPS stimulation, cytokines, IL-18

## Abstract

**Introduction:**

Severe Legionnaires’ disease (LD) can lead to multi-organ failure or death in 10%–30% of patients. Although hyper-inflammation and immunoparalysis are well described in sepsis and are associated with high disease severity, little is known about the immune response in LD. This study aimed to evaluate the immune status of patients with LD and its association with disease severity.

**Methods:**

A total of 92 hospitalized LD patients were included; 19 plasmatic cytokines and pulmonary *Legionella* DNA load were measured in 84 patients on the day of inclusion (day 0, D0). Immune functional assays (IFAs) were performed from whole blood samples collected at D2 and stimulated with concanavalin A [conA, *n* = 19 patients and *n* = 21 healthy volunteers (HV)] or lipopolysaccharide (LPS, *n* = 14 patients and *n* = 9 HV). A total of 19 cytokines (conA stimulation) and TNF-α (LPS stimulation) were quantified from the supernatants. The Sequential Organ Failure Assessment (SOFA) severity score was recorded at D0 and the mechanical ventilation (MV) status was recorded at D0 and D8.

**Results:**

Among the 84 patients, a higher secretion of plasmatic MCP-1, MIP1-β, IL-6, IL-8, IFN-γ, TNF-α, and IL-17 was observed in the patients with D0 and D8 MV. Multiparametric analysis showed that these seven cytokines were positively associated with the SOFA score. Upon conA stimulation, LD patients had a lower secretion capacity for 16 of the 19 quantified cytokines and a higher release of IL-18 and MCP-1 compared to HV. IL-18 secretion was higher in D0 and D8 MV patients. TNF-α secretion, measured after *ex vivo* LPS stimulation, was significantly reduced in LD patients and was associated with D8 MV status.

**Discussion:**

The present findings describe a hyper-inflammatory phase at the initial phase of *Legionella* pneumonia that is more pronounced in patients with severe LD. These patients also present an immunoparalysis for a large number of cytokines, except IL-18 whose secretion is increased. An assessment of the immune response may be relevant to identify patients eligible for future innovative host-directed therapies.

## Introduction

*Legionella* pneumonia, known as Legionnaires’ disease (LD), is an important cause of community-acquired pneumonia (CAP). Although LD is mainly characterized by mild lung dysfunction, it can also progress to multiple organ failure and lead to septic shock (Cecchini et al., 2017; Andrea et al., 2021). The overall mortality rate is approximately 10% but can increase to 30% according to underlying comorbidities and immune status (Chidiac et al., 2012; Cecchini et al., 2017). Non-specific inflammatory mediators have been found to be highly expressed in severe and non-surviving LD patients: C-reactive protein (CRP) level ≥500 mg/L at admission was described as related to mortality in a large cohort (Chidiac et al., 2012) and high serum procalcitonin concentration was associated with higher initial severity score (Haeuptle et al., 2009), intensive care unit (ICU) admission, and hospital death rate (de Jager et al., 2009).

Sepsis is defined as a life-threatening organ dysfunction caused by a dysregulated host response to infection (Singer et al., 2016). The host immune response to sepsis involves a first stage of cytokine overexpression. While some patients with sepsis maintain a robust immune response, a concomitant or following stage of immunoparalysis can induce poor outcomes (higher mortality, longer hospital stay, and secondary hospital infection acquisition) in a subset of patients (Van Der Poll et al., 2021). The latter have altered immune function characterized by low cytokine release after leukocyte stimulation, lymphopenia, and a low expression of monocyte human leukocyte antigen-DR (mHLA-DR), which has been described as a surrogate marker of sepsis-induced immunoparalysis. In particular, an impaired secretion of tumor necrosis factor-α (TNF-α) and other pro-inflammatory cytokines after an *ex vivo* lipopolysaccharide (LPS) challenge, named endotoxin tolerance, has been demonstrated in these patients (Hotchkiss et al., 2013; Delano and Ward, 2016; Van Der Poll et al., 2021).

In the context of COVID-19 or pneumococcal CAP, a hyper-inflammatory profile has been associated with disease severity (Fernández-Serrano et al., 2003; Burgmeijer et al., 2019; Leisman et al., 2020). Altered innate and adaptive immune responses, including severe lymphopenia as well as phenotypic and functional T-cell alterations, have also been described in critically ill COVID-19 patients (Osuchowski et al., 2021; Venet et al., 2021). Data regarding the immune response in LD are still lacking: only two studies have described an increase in T-helper 1 cytokines and interleukin (IL)-6 and IL-8, and no study has assessed the immune functional capacities of LD patients (Tateda et al., 1998; Fernández-Serrano et al., 2003).

The present work aims to assess whether patients display a dysregulated innate or adaptive immune response at the early stage of LD and to study the association between their immune status and the pulmonary injury or overall disease severity.

## Materials and methods

### Study design

This study included LD patients hospitalized in ICUs or conventional medical departments of 12 French hospitals between August 2017 and January 2021. Patients were enrolled within a median of 1 day (interquartile range, IQR [1–3]) after diagnosis. A total of 19 plasmatic cytokines and the pulmonary *Legionella* DNA load were measured at the day of inclusion (day 0, D0) in a group of patients (group A, *n* = 84). Immune functionnal assay (IFAs) were performed at day 2 (D2) in patients hospitalized at the University Hospital of Lyon, separated according to the stimulant used: samples from group B patients (*n* = 19 ICU patients) and from healthy volunteers (HV, *n* = 21) were stimulated with concanavalin A (conA), while samples from group C patients (*n* = 6 ICU and 8 non-ICU patients) and from HV (*n* = 9) were stimulated using LPS. As described in **Figure S1**, LD patients may belong to one or more groups. HV were matched for age and sex. For severity assessment, the Sequential Organ Failure Assessment (SOFA) score and the mechanical ventilation (MV) status were recorded at D0 for all patients; the MV status was recorded at day 8 (D8) for ICU patients only. We assumed that patients (*n* = 22) who were not in the ICU at D8 did not receive MV on D8. At D8, patients were classified using an ordinal severity scale (discharge, ward, ICU, ICU+MV, death) derived from the WHO scale for COVID-19 severity assessment (Marshall et al., 2020). Evidence of septic shock was recorded during ICU stay, based on the sepsis 2 definition: vasopressor requirement to maintain a mean arterial pressure of 65 mm Hg or greater (Singer et al., 2016).

### Ethics and regulatory issues

Adult patients were enrolled in the national prospective cohort “ProgLegio”, which aimed to identify bacterial and human biomarkers of prognostic value for severe LD (NCT03064737; project PRTS ANR/DGOS, ANR-15-CE17-0014NCT03064737), using the following inclusion criteria: (i) patients with clinical and laboratory signs of LD (positive urinary antigen test and/or *L. pneumophila* PCR on respiratory sample), and (ii) having provided written informed consent (legal representative could be used as a surrogate). Exclusion criteria were as follows: (i) LD caused by *Legionella* non *pneumophila*; (ii) patients for whom respiratory secretions could not be obtained, (iii) cases diagnosed only by serology, and (iv) outpatients. Age and immunosuppression status (IS, e.g., long-term corticosteroids, immunosuppressive therapy including anti-TNF-α and other biotherapies, active solid cancer or hemopathy, and other diseases inducing an immunosuppression) were collected for all LD patients but were not considered as exclusion criteria. This study was approved by the regional institutional review board (*Comité de Protection des Personnes Sud-Est IV, France*; ID-RCB 2016-A01021-50). It was registered with the *Ministère de l'Enseignement supérieur, de la Recherche et de l'Innovation* (DC-2008–509) and the French data protection agency (*Commission nationale de l’informatique et des libertés*). Serum samples from HV were obtained from the French national blood services (*Etablissement Français du Sang*, EFS) and used as controls; their written non-opposition to the use of donated blood for research purposes was obtained, according to the EFS standardized procedures.

### *Legionella* pulmonary DNA load

Pulmonary samples (sputum, *n* = 49; tracheal aspirates, *n* = 30; and broncho-alveolar lavages, *n* = 4) were taken at D0 and stored at −20°C before DNA extraction. DNA was extracted from 200 µL of sample using the MagNA Pure Compact Instrument (Roche, Basel, Switzerland) automated system. *Legionella* DNA load was next estimated from 5 µL of DNA sample by a *mip* qPCR as already described (Mentasti et al., 2012). A calibration range was applied using Lp DNA standard reference material for quantification (Baume et al., 2013). Results were expressed in genome units (GU) per reaction and a DNA load >0.1 GU/reaction was considered as positive.

### Cytokines measurement

#### Luminex

Plasma samples and supernatant from whole blood samples after conA stimulation were stored at −20°C before cytokine measurement. A total of 19 circulating cytokines (G-CSF, GM-CSF, IFN-γ, IL-1β, IL-2, IL-4, IL-5, IL-6, IL-7, IL-8, IL-10, IL-12, IL-13, IL-17A, MCP-1, IL-1α, IL-18, MIP-1β, and TNF-α) were simultaneously quantified using a Luminex technology (Bio-Plex 200, BioRad) from thawed plasma and post-conA stimulation supernatant. Results were expressed in pg/mL.

#### Ella automated immunoassay

Supernatant from whole blood samples after LPS stimulation were stored at −20°C before cytokine measurement. TNF-α was quantified from the thawed supernatant on Simple plex cartridges (ProteinSimple, San Jose, CA) using the Ella nanofluidic system (Biotechne, Minneapolis, MN), according to the manufacturer’s instructions. Results were expressed in pg/mL.

### Immune functional assays

IFAs were performed in heparinized whole blood from patients of groups B and C sampled at D2 and from HV; stimulation of white blood cells (WBCs) occurred within 3 h after whole blood collection. For group B patients (*n* = 19) and matched HV (*n* = 9), 500 µL of fresh blood was incubated for 24 h at 37°C with a Type IV conA. The latter acts as a strong and nonspecific activator of T-cell apoptosis and autophagy by binding osidic residues at the surface of lymphocyte T-cell receptors (concentration= 2.5 mg/mL, Sigma-Aldrich, St. Louis, MO, USA). For group C patients (*n* = 14) and matched HV (*n* = 9), 1 mL of blood was incubated for 24 h at 37°C in standardized TruCulture tubes (Myriad Rbm, Austin, TX, USA) prefilled with ultrapure *E. coli* LPS (100 ng/mL, *E. coli* O55:B5). The latter is a toll-like receptor (TLR)-4 and -2 ligand, which triggers the innate immunity and induces an intense cytokine and chemokine secretion and the maturation of antigen presentation. In parallel, a negative control (NUL condition) containing only culture medium (Gibco RPMI 1640 medium, Fisher Scientific SAS, Illkirch, France) was carried out for all patients and HV samples.

### mHLA-DR measurement

Expression of surface mHLA-DR was measured by flow cytometry from EDTA samples collected within 2 h at D2 or D3 for 18 patients belonging to group B. The anti-HLA-DR/Anti-Monocyte Quantibrite assay (BD Biosciences, San Jose, USA) was performed on a Navios flow cytometer and data were analyzed using Navios software (NAVIOS; Beckman-Coulter, Brea, CA, USA). Monocytes were gated based on CD14 expression. mHLA‐DR expression was measured as the median of fluorescence intensity related to the entire monocyte population, as recommended by the manufacturer (Demaret et al., 2013). The fluorescence was converted to antibodies bound per cell (Ab/C) using a calibrated standard curve determined with phycoerythrin (PE)‐beads (BD QuantiBrite™ ‐ PE Beads, Becton Dickinson). Results are expressed as number of sites per cell (Ab/C). The usual values are 13,500–45,000 Ab/C and the threshold value for immunosuppression is <8,000 Ab/C (Venet et al., 2022).

### Statistical analyses and cluster building

Results are expressed as median and IQR for continuous variables. Statistical analyses were conducted using GraphPad Prism Software 8 (San Diego, CA, USA). Non-parametric Mann–Whitney tests were used for rank comparisons between two unpaired groups, and non-parametric Kruskal–Wallis tests were used for comparisons between three or more paired or unpaired groups. Unpaired *t*-tests with Welch’s correction were used to compare the means of mHLA-DR expression and post-LPS TNF-α secretion levels. *R*² (eta-squared) coefficients were calculated to measure the effect size between patients and normal value, patients and HV values, and D0 and D8 MV status. The effect size was considered small for 0.01 ≤ *r*² ≤ 0.09, medium for 0.09 ≤ *r*² ≤ 0.25, and large for *r*² > 0.25. Non-parametric chi-squared tests were used for proportion comparisons between groups A, B, and C, and among the entire cohort for MV/no-MV comparisons. Principal component analysis (PCA) was carried out using the PCA formula from FactoMineR package (R software version 3.5.1). A heatmap was generated by scaling and centering log10-transformed cytokines concentrations, and the dendogram was drawn based on hierarchical clustering analysis (Euclidean distance matrix with Ward’s method) using the heatmap3 package (R software version 3.5.1). A *p*-value <0.05 was considered significant.

## Results

### Patient characteristics

A total of 92 patients from the ProgLegio trial were enrolled. D0 MV patients (*n* = 36) were more severe than no-MV patients (*n* = 56). The D0 MV patients were more often admitted to the ICU; had a higher median SOFA score and a lower median blood pressure; were more frequently under vasopressors, hemofiltration, and MV at D8; more frequently developed septic shock during their stay; and had longer hospital and ICU stays (**Table 1**). D0 MV patients had higher WBC count, lower lymphocyte count, and higher pulmonary *Legionella* DNA load. In contrast, the proportion of LD risk factors, including immunosuppression, and the D28 mortality rate were not significantly different between D0 MV and no-MV patients. A higher proportion of D0 MV patients received a combination therapy for LD (fluoroquinolones and macrolides; **Table 1**), but there was no significant difference in combination therapy administration according to the inclusion hospital.

**Table 1:** Clinical and laboratory data of LD patients according to D0 mechanical ventilation status.

Criteria	LD patients (*n* = 92)	No-MV (*n* = 56)	MV (*n* = 36)	*p*-value
LD risk factors				
Smoking, *n* (%)	45 (49)	27 (48)	18 (50)	1
COPD, *n* (%)	9 (10)	5 (9)	4 (11)	0.73
Alcoholism, *n* (%)	9 (10)	3 (5)	6 (17)	0.15
Diabetes, *n* (%)	10 (11)	4 (7)	6 (10)	0.18
Immunosuppression, *n* (%)	25 (27)	14 (56)	11 (31)	0.63
Immunosuppressive therapy, *n* (%)	16 (17)	11 (20)	5 (13)	0.42
- corticosteroids, *n* (%)	12 (13)	9 (16)	3 (8)	0.35
- other, *n* (%)	3 (3)	1 (2)	2 (6)	0.56
Cancer/hemopathy, *n* (%)	5 (5)	3 (5)	2 (6)	1
Other immunosuppressive conditions, *n* (%)	6 (7)	3 (5)	3 (8)	0.68
≥ 1 risk factor	69 (75)	42 (75)	27 (75)	1
Inclusion data (D0)				
ICU admission, *n* (%)	60 (65)	24 (43)	36 (100)	**<0.0001**
SOFA score, median [IQR]	3 [1–7]	1 [0–3]	8 [4–9.8]	**<0.0001**
Fluoroquinolone and macrolide combination therapy (*n* = 81), *n* (%)	57 (70)	26 (54)	31 (94)	**0.0001**
D0 laboratory parameters				
White blood cells (G/L), median [IQR]	12.7 [8.9–15.8]	11.2 [8.4–15]	14.2 [10.0–21.9]	**0.04**
Polynuclear neutrophils (G/L), median [IQR]	10.8 [7.9–14.6]	10 [7.1–13]	12.7 [8.5–20]	0.06
Lymphocytes (G/L), median [IQR]	0.80 [0.48–1.1]	0.93 [0.64–1.23]	0.55 [0.35–1.13]	**0.03**
CRP (mg/L), median [IQR]	310 [197–421]	296.7 [207–418.3]	309 [185.3–392.3]	0.96
Creatininemia (µmol/L), median [IQR]	87.5 [71.5–160.3]	81 [69–114]	97 [73–203]	0.06
Pulmonary *Legionella* DNA load (GU/reaction), median [IQR]*	48.5 [0.9–731.2]	12.86 [0.90–89.9]	455.8 [31.24–7,460]	**0.0006**
Clinical data				
Mean blood pressure (mmHg), median [IQR]	100 [87–110]	103.8 [89.63–111.5]	92.50 [84–105]	**0.01**
Temperature (T°C), median [IQR]	39 [38.5–40]	39 [38–40]	39.9 [39–40]	0.19
Intensive care during ICU stay (*n* = 60)				
Vasopressor, *n* (%)	20 (22)	2 (4)	18 (50)	**<0.0001**
Hemofiltration, *n* (%)	8 (9)	1 (2)	8 (22)	**0.002**
Corticosteroids (sepsis management), *n* (%)	8 (9)	5 (9)	3 (8)	1
Evolution				
Septic shock, *n* (%)	21 (23)	0 (0)	20 (56)	**<0.0001**
D8 MV, *n* (%)	23 (25)	0 (0)	23 (35)	**<0.0001**
Hospitalization duration (d), median [IQR]	10 [6–26]	7.5 [5–10.75]	20 [10–31]	**<0.0001**
ICU duration (d), median [IQR]**	10 [5–16]	5 [2–7.25]	13.5 [8.5–25.75]	**<0.0001**
D28 mortality, *n* (%)	4 (4)	1 (2)	3 (8)	0.29

COPD, chronic obstructive pulmonary disease. Alcoholism corresponds to an alcohol consumption >3 glasses per day for men and >2 glasses per day for women for at least 1 year. Other immunosuppressive therapies include anti-TNFα therapy (Etanercept, Infliximab, Certolizumab, and Adalimumab) and anti-inflammatory biotherapy (Methotrexate). Other immunosuppressive conditions included rheumatoid polyarthritis, solid organ transplantation, and psoriatic arthritis.

MV, mechanical ventilation; IQR, interquartile range; ICU, intensive care unit; LD, Legionnaires’ disease; SOFA, Sequential Organ Failure Assessment; CRP, C-reactive protein; GU, genome unit. The p-value column indicates the p-values of Mann–Whitney tests used for rank comparisons between MV and no-MV patients.

*no data n = 9.

**n = 58.

Inclusion data, D0 laboratory data, ICU stay data, and outcomes were collected.

Bold values are statistically significant results, and normal values (non-bold) are non significant.

**Figure 1:**
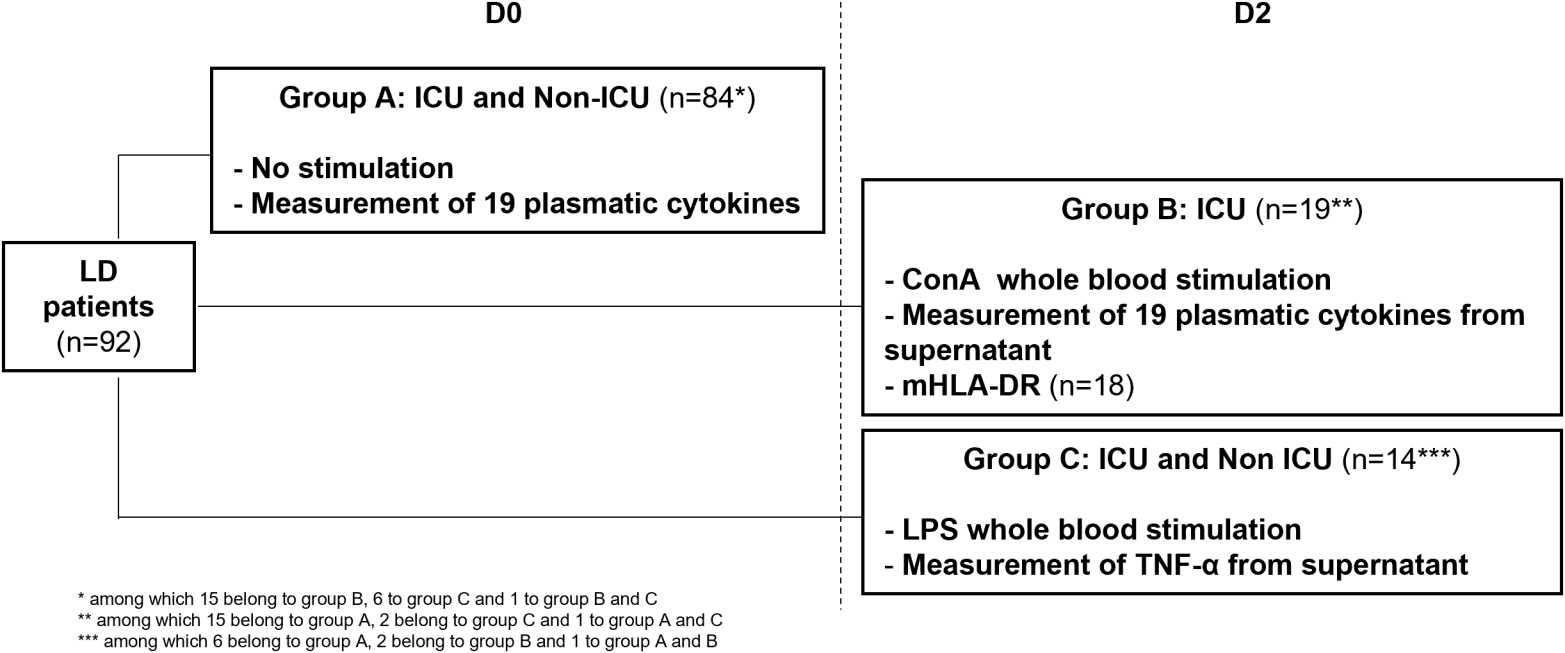
Study design and measurement of immune parameters according to patient group. LD, Legionnaires’ disease; ICU, intensive care unit; ConA, Concanavalin A; mHLA-DR, monocytic human leukocyte antigen DR; LPS, lipopolysaccharide.

Different immune parameters were evaluated in three subgroups of patients (**Figure 1**; **Figure S1**): A (*n* = 84), B (*n* = 19), and C (*n* = 14). Most demographics, LD risk factors (including immunosuppression), clinical, and laboratory parameters were common between groups A, B, C, and the entire cohort (**Table S1**). However, patients in group B, who were only ICU patients, had a higher D0 SOFA score and were more often under MV at D8 compared to groups A, C, and the entire cohort.

### Initial hyper-inflammatory profile in severe patients

**Figure 2:**
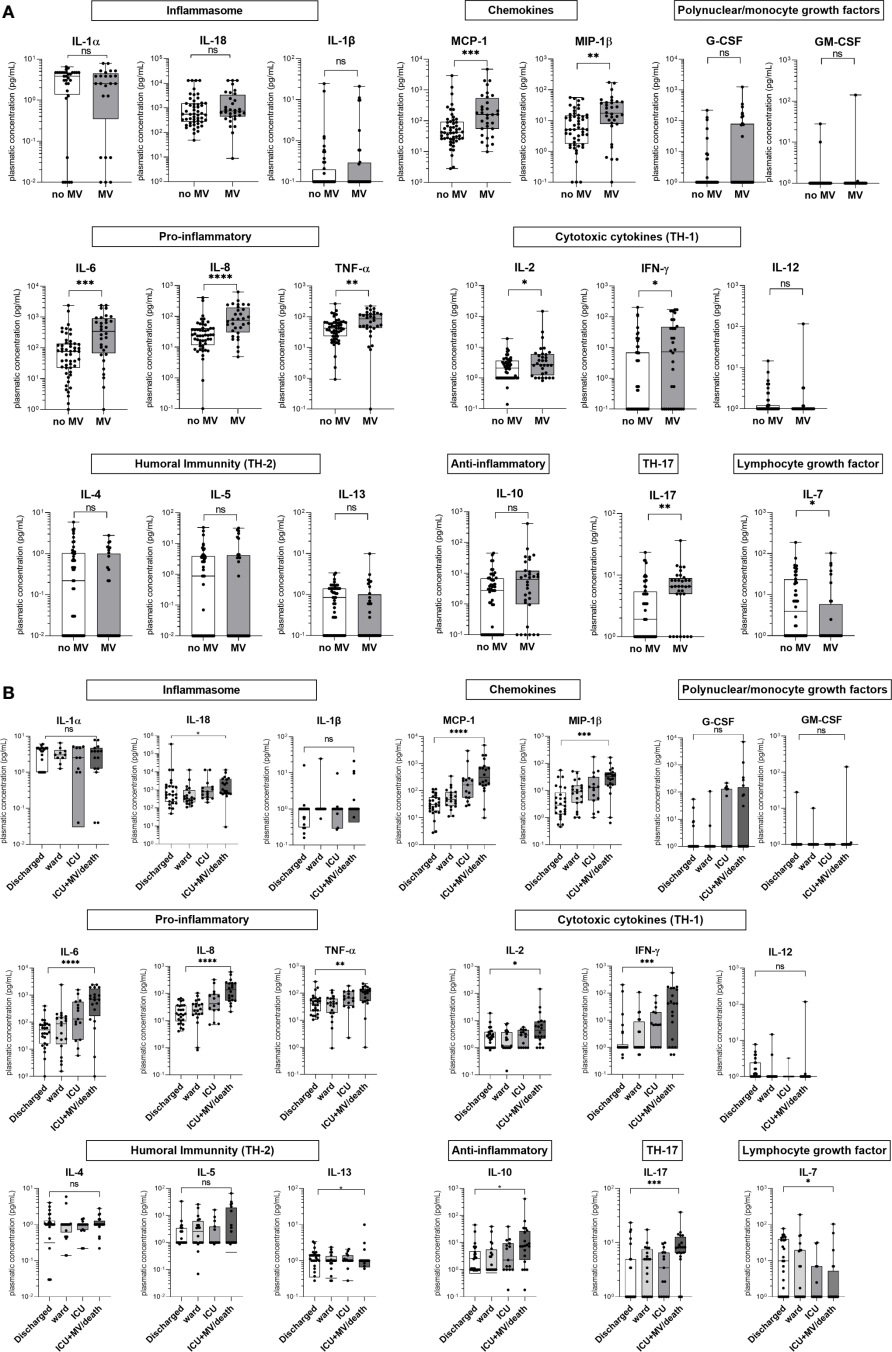
Concentrations of 19 plasmatic cytokines for group A patients at D0 according to mechanical ventilation (MV) status at D0 and to a severity scale at D8. **(A)** Concentrations for D0 MV (*n* = 36) vs. D0 no-MV (*n* = 48). **(B)** Concentrations at D8 according to severity: discharged (*n* = 26), ward (*n* = 20), ICU (*n* = 15), and ICU+MV/death (*n* = 20). We assumed that patients who were not in the ICU at D8 (*n* = 22) did not receive MV at D8. The patient who died between D0 and D8 was excluded from the analysis. Data are represented as boxplots illustrating the median, interquartile range, and range; Mann–Whitney **(A)** or Kruskal–Wallis **(B)** comparison tests: ns, non significant; *p < 0.05, **p < 0.01, ***p < 0.01, ****p < 0.0001.

We first measured 19 plasmatic inflammatory markers in patients from group A at D0 and compared their median expression between patients with (*n* = 32) or without (*n* = 52) MV at D0. Pro-inflammatory cytokines (IL-6, IL-8, and TNF-α), chemokines (MCP-1 and MIP1-β), T-helper (Th)-1 (IL-2 and IFN-γ), and Th-17 (IL-17) cytokines were significantly overexpressed in MV vs. no-MV patients (**Figure 2A**). In contrast, levels of IL-7, a lymphocyte growth factor, was reduced in MV patients. Cytokine levels did not differ significantly according to IS status, underlying immunosuppressive therapy, and corticosteroid administration for sepsis management. The median *Legionella* pulmonary DNA load was higher in D0 MV vs. no-MV patients (455.8 [31.24–7,460] GU/reaction vs. 12.86 [0.90–89.93] GU/reaction, *p* < 0.0001) and D8 MV vs. no-MV patients (1,357 [171.8–25,328] GU/reaction vs. 14.28 [0.835–126.5] GU/reaction, *p* < 0.0001; **Figure S2**).

The cytokine levels were then assessed according to the ordinal severity scale (discharge, ward, ICU, ICU+MV/death) at D8. There was a significant difference in IL-6, IL-8, TNF-α, MCP-1, MIP-1β, IL-17, IFN-γ, IL-2, and IL-18 secretion levels according to severity (**Figure 2B**).

**Figure 3:**
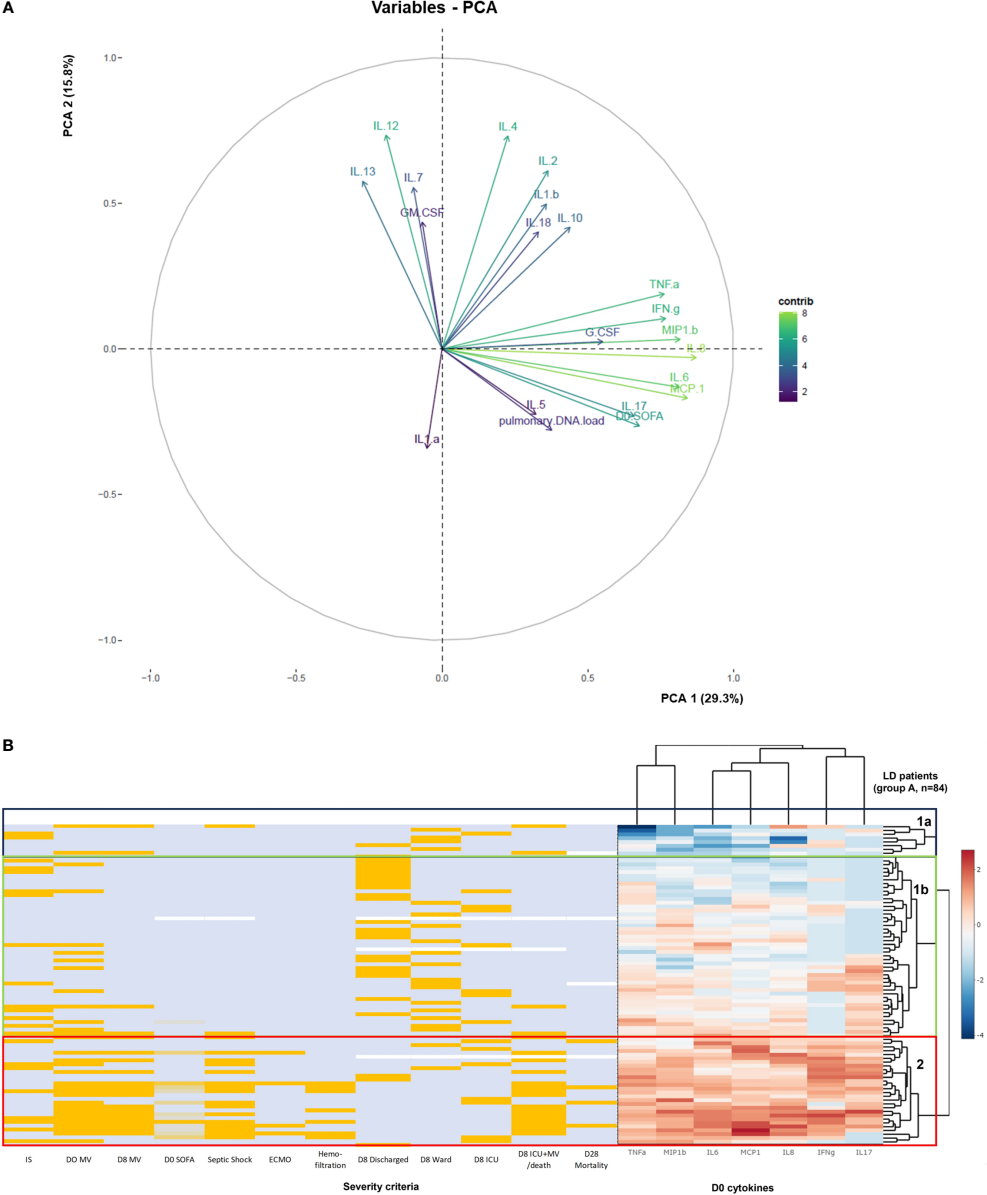
Analysis of inflammatory response in association with *L. pneumophila* DNA and clinical severity criteria in patients from group A. **(A)** Principal component analysis (PCA) performed with 19 cytokines, D0 SOFA score, and pulmonary *Legionella* DNA load. Principal component 1 and 2 (PC1 and PC2) contributed to 29.3% and 15.8% of the variation, respectively. The contribution of the variables to axes 1 and 2 is indicated by the color gradient of the arrows. **(B)** The heatmap of cytokine expression profiles from the supervised analysis (Euclidean distances matrix with Ward’s methods) was generated by scaling and centering the log10-transformed of the values of the seven D0 cytokines that significantly contributed to axis 1. Immunosuppression status, D0 and D8 mechanical ventilation (MV) status, D0 SOFA score, septic shock, ECMO, hemofiltration, severity scale, and the D28 mortality were added. Patients with positive results for previous criteria are shown in orange and patients with negative results are shown in light blue. For SOFA score, light blue corresponds to SOFA ≤ quartile 1 (Q1, SOFA = 1), light orange corresponds to Q1 < SOFA ≤ median (SOFA = 3), and orange corresponds to SOFA > median. Missing values (*n* = 1 for D0 SOFA score and septic shock, *n* = 3 for the severity scale, *n* = 4 for D28 mortality) are shown in white. Blue box corresponds to cluster 1a, green box corresponds to cluster 1b, and red box corresponds to cluster 2.

A PCA was next performed in patients from group A to visualize the expression of plasmatic cytokine secretion, pulmonary *Legionella* DNA load, and D0 SOFA score using a single representation (**Figure 3A**). The variables projected onto the first two principal components showed an overall variance of 45.1%. Eigenvalues and individual values are presented in **Figure S3**. A main group of seven cytokines (MCP-1, MIP1-β, IL-6, IL-8, TNF-α, IL-17, and IFN-γ) in addition to the D0 SOFA score contributed the most to axis 1 (29.3%). In contrast, IL-12, IL-2, and IL-4 formed a smaller group, which mostly contributed to axis 2 (15.8%). Other cytokines (IL-13, IL-7, IL-1β, IL-10, GM-CSF, G-CSF, IL-1α, and IL-5) and the pulmonary DNA load did not appear to have a major contribution to axis 1 or 2.

We then used an unsupervised hierarchical clustering to assess the profile of each patient according to the expression of the seven cytokines contributing to axis 1, IS, D0 and D8 MV status, D0 SOFA score, septic shock, Extracorporeal Membrane Oxygenation (ECMO) and hemofiltration status, the severity scale, and the D28 mortality (**Figure 3B**). The heatmap divided the patients into two clusters (1, *n* = 56 and 2, *n* = 28) and two subgroups within cluster 1 (1a, *n* = 8 and 1b, *n* = 48). Patients from cluster 2 had high cytokine secretion compared to patients from cluster 1 who had low (cluster 1a) or intermediate (cluster 1b) cytokine expression. While IS status was not associated with any cluster, 64% (18/28) of patients from cluster 2 had a D0 MV, and 54% (15/28) had a D8 MV compared to 25% (14/56) and 9% (5/56), respectively, for patients from cluster 1. In cluster 2, 54% (15/28) of patients had septic shock compared to 5% (3/56) in cluster 1 (*p* < 0.0001). Overall, 82% (23/28) of the patients from cluster 2 were D8 ICU or D8 ICU + MV/death according to the severity scale. The median D0 SOFA score was higher in patients from cluster 2 compared to cluster 1 (8 [3–10] vs. 2 [0–4], *p* < 0.0001). Cluster 2 also grouped all ECMO patients (*n* = 4), all patients with hemofiltration (*n* = 7), and all D28 non-survivors (*n* = 4). These results suggest that patients with non-severe LD (cluster 1) may be differentiated from patients with severe LD (cluster 2) based on their cytokine profile.

### Impaired cytokine secretion post-conA stimulation in LD patients

**Figure 4:**
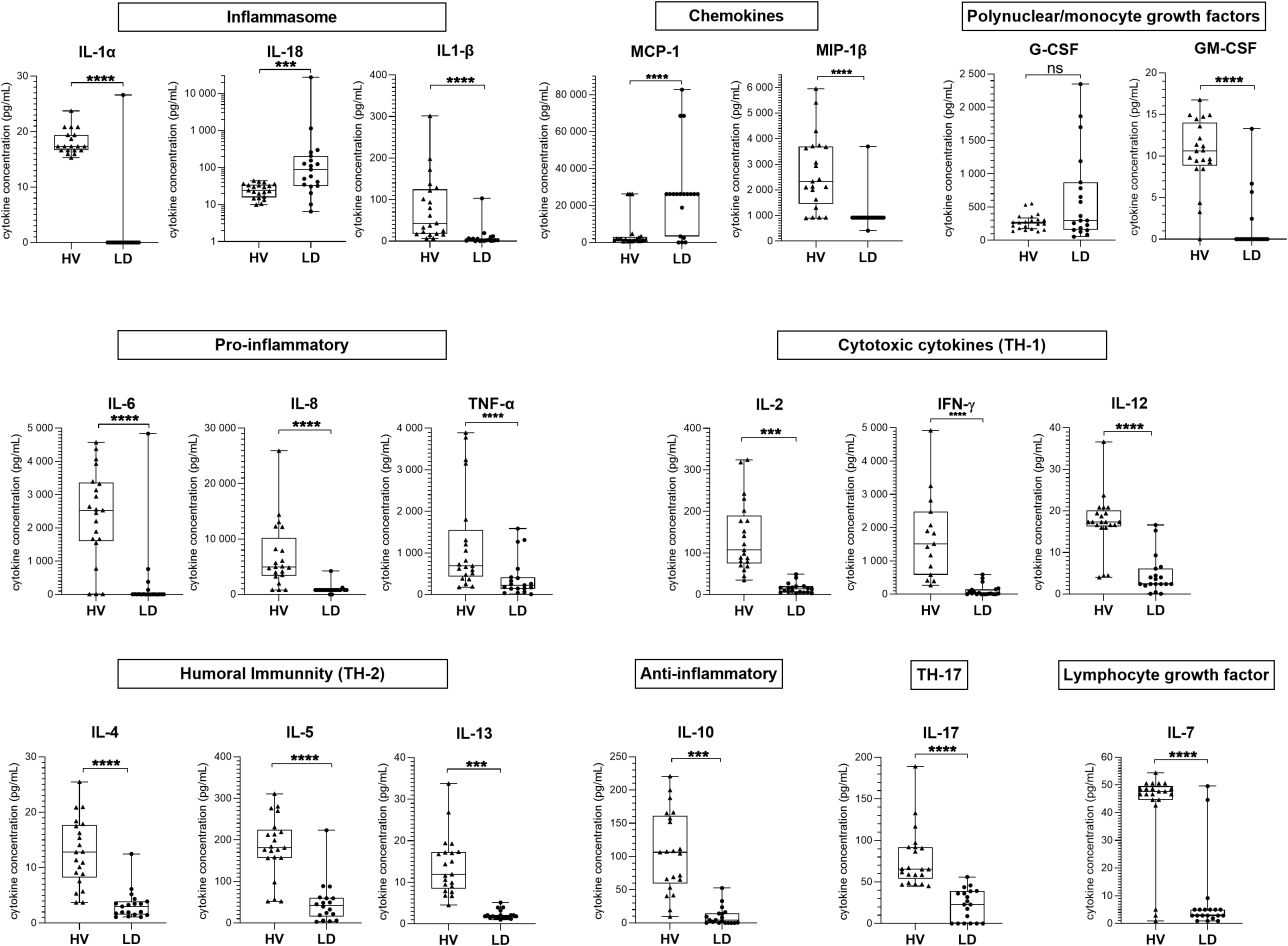
Cytokine concentration in supernatant after conA whole blood stimulation in patients from group B compared to healthy volunteers. The supernatant concentration of 19 cytokines from samples of group B patients (LD, *n* = 19) compared to those of healthy volunteers (HV, *n* = 21), after stimulation with conA, are shown. Data are represented as boxplots illustrating the individual values, median, interquartile range, and range; Mann–Whitney comparison tests: ns, non significant; ***p < 0.01, ****p < 0.0001.

To study the immune functional response of LD patients in relation to disease severity, we performed an IFA at D2 in patients from group B (*n* = 19 ICU patients). Among them, 32% (6/19) had a septic shock during their stay (**Table S1**). We compared WBC function between LD patients and HV (*n* = 21) after stimulation with the non-specific lymphocyte activator conA. A total of 16 out of 19 mediators were significantly less released in LD patients (**Figure 4**): IL-1α and IL-1β inflammasome cytokines, MIP-1β chemokine, pro-inflammatory cytokines (IL-6, IL-8, and TNF-α), GM-CSF growth factor, Th1 (IL-2, IFN-γ, and IL-12), Th2 (IL-4, IL-5, and IL-13), and Th17 (IL-17) cytokines, IL-7 lymphocyte growth factor, and IL-10 anti-inflammatory cytokine. In contrast, the inflammasome cytokine IL-18 and the chemokine MCP-1 were increased. The cytokine levels did not differ significantly according to IS status. As LD patients had a higher leukocyte count (median [IQR]: 12.8 [6.9–15.0] vs. 6.4 [5.3–7.2], *p* = 0.0015) and a lower lymphocyte count (median [IQR]: 0.97 [0.40–1.75] vs. 1.82 [1.62–2.32] G/L, *p* = 0.0010) than HV, the cytokine concentration was normalized to the lymphocyte count. After normalization, the secretion of 16/19 mediators still varied in the same way, but the secretion of TNF-α and MIP-1β was no longer significantly different between HV and LD and G-CSF was increased in LD.

We next assessed whether the low leukocyte response was associated with the severity of the disease, expressed by D0 or D8 MV. There was no significant difference between severe and non-severe group B patients according to D0 MV status for 18 of the 19 measured parameters (**Figures S4A**, **S4B**). However, the median IL-18 secretion was higher in patients with a D0 MV (126.9 [69.3–279.1] vs. 25.5 [9.2–40.4] pg/mL, *p* = 0.0009, **Figure S4A**) and in those with a D8 MV (88.7 [36.6–650.6] vs 30.6 [10.1–58.2] pg/mL, *p* = 0.029, **Figure S4B**) than in no-MV patients. The secretion variations had a similar pattern when using hemofiltration or septic shock as severity criteria.

### mHLA-DR expression compatible with an immunoparalysis status in ICU patients

Furthermore, we measured the expression of mHLA-DR at D2 or D3 in 18 patients from group B. There was a medium effect size in the mean ± SD mHLA-DR expression between the patients and the normal value (difference between means −3,447 ± 7,856 Ab/C, 95% CI [−6,690; −204], *r*² = 0.17), suggesting an immunosuppressed status for these patients. Among the 18 patients, 72% (13/18) had MV at D0 and 61% (11/18) had MV at D8. There was a medium effect size in mHLA-DR expression between D0 MV and D0 no-MV patients (−5,692 ± 7,155 Ab/C, 95% CI [−12,847; 1,464], *r*² = 0.17) and a small effect size for D8 MV status (−4,044 ± 6,434 Ab/C, 95% CI [−10,478; 2,390], *r*² = 0.07).

### Low TNF-α secretion post-LPS stimulation in patients with D8 MV

**Figure 5:**
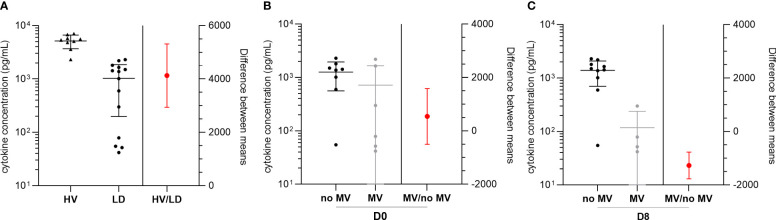
TNF-α concentration after LPS stimulation at D2 in the supernatant of group C patients according to mechanical ventilation (MV) status. **(A)** Mean TNF-α concentration in the supernatant of group C patients (*n* = 14) compared to healthy volunteer (HV) samples (*n* = 9) after stimulation with LPS and difference between means. TNF-α concentration in LD patients and differences between means according to D0 **(B)** and D8 **(C)** MV status. Data plotted on the left *Y* axis are represented as scatter dot plots illustrating the individual values, mean, and SD. Data plotted on the right *Y* axis are represented as mean and SD.

We next assessed whether the post-stimulation low cytokine secretion could also be observed in patients with less severe LD. For this purpose, we stimulated whole blood collected at D2 from group C patients (*n* = 6 ICU patients and *n* = 8 non-ICU patients) in TruCulture tubes prefilled with LPS and compared their immune response with that observed in 9 HV. Among LD patients, 14% (3/14) had a septic shock during their stay (**Table S1**). There was a large effect size in the mean ± SD TNF-α secretion between HV and LD patients (4,125 ± 1,186 pg/mL, 95% CI [2,939; 5,311], *r*² = 0.84; **Figure 5A**). The effect size was medium between D0 no-MV and D0 MV (542.0 ± 1,043 pg/mL, 95% CI [−500.9; 1,585], *r*² = 0.14; **Figure 5B**) but large between D8 no-MV and D8 MV (−1,273 ± 501.8 pg/mL, 95% CI [−1,775; −771.2], *r*² = 0.76; **Figure 5C**) The effect size was also large between patients without and with septic shock (−1,234 ± 491.6, 95% CI [−1,726; −742.6] pg/mL, *r*² = 0.76). The TNF-α levels did not differ significantly according to the IS status.

## Discussion

The study herein showed that LD patients with D0 MV had a higher systemic pro-inflammatory burst. Furthermore, at D2, after non-specific whole blood stimulation, most cytokine secretion capacities were significantly reduced in LD patients compared to HV, suggesting a leukocyte hyporesponsiveness. Interestingly, a standardized IFA with LPS underlined that the patients with the lowest TNF-α release were the ones still under MV at D8.

The present results add original data on the immune response of LD patients, highlighting an initial phase of intense pro-inflammatory mediator expression in the most severe patients. Using multi-parametric analysis, the initial SOFA score was associated with a profile of seven pro-inflammatory parameters, i.e., three pro-inflammatory cytokines (IL-6, IL-8, and TNF-α), two chemokines (MCP-1 and MIP1-β), Th-1 (IL-2 and IFN-γ), and Th-17 (IL-17) cytokines. In contrast, pulmonary *Legionella* DNA load was less associated with SOFA score than these seven cytokines. Hierarchical clustering confirmed that the severity criteria (D0 and D8 MV and D0 SOFA score) were more present in a cluster of patients with initial high levels of the cytokines previously mentioned. This suggests that the severity of LD may be related to hyper-inflammation. The immune response following *Legionella* infection has mainly been studied in cellular and animal models. TNF-α, highly secreted *in vitro* and in mice models (McHugh et al., 2000; Guillemot et al., 2022), has been demonstrated to be a key cytokine for the bacterial restriction (Fujita et al., 2008). Elevated concentrations of IFN-γ and IL-6 are also found in murine models (Spörri et al., 2006; Liu et al., 2020). The present results are consistent with previous findings showing a high expression of circulating Th-1 cytokines, IL-6, and IL-8 in LD patients (Tateda et al., 1998; Fernández-Serrano et al., 2003) and complete the description of the immune profile in a larger cohort of patients. The systemic overexpression of inflammatory cytokines has also been described in other severe infectious pneumonia such as COVID-19 and pneumococcal or *Mycoplasma pneumoniae* CAP (Fernández-Serrano et al., 2003; Burgmeijer et al., 2019; Leisman et al., 2020; Hu et al., 2021), in which the excessive inflammation has been shown to lead to fatal tissue damage in the lung (Xu et al., 2023). In the present study, severe LD patients had a more pronounced lymphopenia, as previously described in LD, septic shock, and severe COVID-19 patients (de Jager et al., 2013; Girardot et al., 2017; Bermejo-Martin et al., 2020). Absolute lymphopenia may result from T-cell migration to the lungs and increased apoptosis mediated by the high levels of circulating pro-inflammatory cytokines (de Jager et al., 2013; Girardot et al., 2017).

Furthermore, the present study shows that leukocytes in LD patients are impaired in their ability to release cytokines within the first days after infection. The IFA with in-house T-cell stimulation using conA showed a significantly lower production of 16 cytokines from major innate and adaptive pathways compared to HV. These alterations were observed in severe and non-severe patients, regardless of the development of a septic shock during the hospital stay. The exception are IL-18 and MCP-1, whose production was increased upon *ex vivo* stimulation of patient WBC compared to HV. IL-18 production suggests that the caspase-1-dependent inflammasome pathway leading to the release of IL-18 is functional. In animal models of *Legionella* infection, it has indeed been proven that the NLR family CARD domain containing 4 (NLRC4) inflammasome drives pyroptosis through caspase-1 activation and IL-18 release (Case et al., 2009; Mascarenhas and Zamboni, 2017). IL-18 expression was the only elevated cytokine in patients with initial MV or an unfavorable ventilatory evolution. While no study has assessed IL-18 secretion in human LD, several studies have shown that high plasma IL-18 levels are associated with poor clinical outcome in patients with bacterial sepsis (El-Sayed Zaki et al., 2007; Feng et al., 2016). Moreover, IL-18 mRNA transcript was recently shown to be overexpressed in a cohort of patients with septic shock (Tawfik et al., 2020).

Nowadays, the use of IFA with standardized stimulant concentrations has been shown to provide robust and reproducible results (Albert-Vega et al., 2018; Mouton et al., 2020). In order to standardize IFA and obtain a stimulation of innate immune cells complementary to the one obtained with conA, we used commercial TruCulture tubes prefilled with a fixed concentration of stimulant. *Escherichia coli* LPS was selected due to its ability to induce a strong activation of innate and adaptive immunity in HV (Urrutia et al., 2016) and because it is the reference test for endotoxin tolerance (Biswas and Lopez-Collazo, 2009). As most inflammatory mediators were underexpressed following conA stimulation, we measured only one representative parameter for this standardized IFA. TNF-α was chosen because it is one of the surrogate markers of endotoxin tolerance and plays a key role in *Legionella* restriction. Through this assay, we confirmed the quantitative defect in the production of TNF-α in LD patients compared to HV. In the context of sepsis, the post-LPS stimulation cytokine release, or endotoxin tolerance, is part of the sepsis immunoparalysis (Hotchkiss et al., 2013; Albert Vega et al., 2020). A study showed a lower TNF-α response to LPS in patients with sepsis or septic shock compared to less severe post-surgical patients (Winkler et al., 2017). In line with these findings, we showed a lower TNF-α release capacity for D8 MV compared to no-MV patients. Furthermore, ICU LD patients also displayed monocyte dysfunction with weak expression of mHLA-DR, suggesting that these patients share features of sepsis-induced immunosuppression (Hotchkiss et al., 2013; Venet and Monneret, 2018). Interestingly, LPS stimulation allowed us to identify that patients with MV at D8 had a lower TNF-α secretion at D2. However, since all D8 MV patients were under MV at D0, we could not assess whether the impaired TNF-α release was a predictor or rather a consequence of MV. Conversely, we did not show any difference with conA stimulation between MV and no-MV. This could be explained by the different pathways activated by each stimulant: conA induces a strong and non-specific activation of T-cells whereas LPS mimics a bacterial infection by activating TLR-4 and TLR-2 innate receptors. The *in vitro* LPS stimulation may thus be closer to the *in vivo Legionella* infection (Akamine et al., 2005).

The present study has several limitations. Patients were enrolled based on positive Lp1 urinary test, and therefore, patients with non-Lp1 LD [<20% of cases in Europe and the USA (Beauté and Network, 2017)] were not included. In addition, the ICU admission rate was higher than previously described (Levcovich et al., 2016) which could lead to an over-representation of severe LD and thus increase the observed cytokine variations. Patient WBC count, and particularly lymphopenia, could represent an important confounder for IFA. Although a rather similar pattern of cytokine secretion was shown after normalization to lymphocyte blood counts, some changes were observed. Moreover, this normalization could not be performed for the entire cohort. Another limitation relates to the methodological issue of requiring fresh whole blood collection for IFA and mHLA-DR measurements, allowing these parameters to be tested only for a limited number of patients, in two separate cohorts, hospitalized in the same hospital. This pre-analytical requirement complicates the routine use of such immunosuppression markers. The small number of subjects tested may have reduced the robustness of the findings regarding the association with severity, and has precluded the classification of patients according to the hard endpoint (e.g., the severity scale). Furthermore, the association between baseline cytokine release and impaired antigen-induced response is well described in sepsis and severe COVID-19 infection; the small number of patients tested herein using IFA, however, did not allow to evaluate such an association. Finally, since only TNF-α was measured using a standardized IFA, the release of other Th-1 and pro-inflammatory mediators known to be impaired in sepsis, as well as mHLA-DR expression, need to be evaluated using a similar assay in a larger multicenter LD cohort.

A study by Lettinga et al. showed that LD patients from a large outbreak maintained low IFN-γ capacity secretion following *E. coli* LPS stimulation up to 1 year after the initial infection compared to exposed but uninfected individuals (Lettinga, 2003). These data suggest either that low function may persist for some time or that a basal low cytokine production capacity may contribute to the susceptibility to *Legionella* infection. Whether these alterations persist over time in the month following the infection should be assessed. LD patients show profound alterations in TNF-α secretion after LPS stimulation, the degree of alteration being related to disease severity as it has been described in sepsis. Immunostimulatory therapies (IFN-γ and GM-CSF) have been attempted in clinical trials for septic shock (Hotchkiss et al., 2013). More recently, IL-7 immunostimulation has shown functional recovery of lymphoid cells and a reduced mortality in cellular and animal models (Venet et al., 2012; Shindo et al., 2017). Therapeutical trials showed an increase in T-cell proliferation in septic shock patients treated with IL-7 compared to placebo (Francois et al., 2018; Daix et al., 2023). As LD patients have comparable immune defects to septic shock patients, such treatment could be proposed as a personalized medicine after routine assessment of sepsis-induced IS.

In conclusion, we describe a hyper-inflammatory phase at the initial phase of *Legionella* pneumonia that is more pronounced in patients with severe LD. These patients also present an immunoparalysis for a large number of cytokines, except IL-18 whose secretion is increased. An assessment of the immune response may be relevant to identify patients eligible for future innovative host-directed therapies.

## Data availability statement

The raw data supporting the conclusions of this article will be made available by the authors, without undue reservation.

## Ethics statement

The studies involving humans were approved by Comité de Protection des Personnes Sud-Est IV, France; ID-RCB 2016-A01021-50. The studies were conducted in accordance with the local legislation and institutional requirements. The participants provided their written informed consent to participate in this study.

## Author contributions

The project was conceived, planned, and supervised by SJ. SJ, FA, CA, GD, LB, MI, PD, JG, AC, and GL were involved in the design, implementation, and day-to-day management of the study. WM and ST-A were involved in the IFA selection and analysis of the results. CA-V brought post-LPS stimulation HV data. J-CR and FA brought their expertise about clinical and biological data interpretation. LA, J-CR, AF, VL, YJ, NaF, and FA included patients in the study. FV was responsible for the mHLA-DR generation. CA, HT, NoF, GD, LB, MI, and SJ were responsible for the microbiological analyses; CA and NaF were responsible for the immunological analyses. CA was involved in the statistical analyses. CA and SJ wrote the original draft of the manuscript, which was reviewed and edited by WM, ST-A, FV, JG, PD, AC, and FA and reviewed by all co-authors. All authors approve the final version of the manuscript.
