# Supplementary material for: Hyper-inflammatory profile and immunoparalysis in patients with severe Legionnaires’ disease
Source: Front Cell Infect Microbiol. 2023 Oct 27;13:1252515. doi: 10.3389/fcimb.2023.1252515 (PMC10641404; doi:10.3389/fcimb.2023.1252515)
Supplement: Supplementary file 1 [file DataSheet_1.docx]

**Table S1. Characteristics of all LD patients and according to groups A, B, and C.** Demographics, LD risk factors, admission data, D0 laboratory data, ICU stay data, and outcomes were collected.

| **Criteria** | | **LD patients**  **(n=92)** | **Group A**  **(n=84)** | **Group B**  **(n=19)** | **Group C**  **(n=14)** | **p-value** |
| --- | --- | --- | --- | --- | --- | --- |
| **Demographics** |  | |  |  |  |  |
| Male sex, n (%) | 66 (72) | | 60 (71) | 12 (69) | 11 (79) | 0.80 |
| Age (y), median [IQR] | 64.5 [56-72.3] | | 63.5 [55.3-73] | 66 [56-71] | 62 [53-70] | 0.84 |
| **LD risk factors** |  | |  |  |  |  |
| Smoking, n (%) | 45 (49) | | 42 (50) | 10 (53) | 4 (29) | 0.49 |
| COPD, n (%) | 9 (10) | | 8 (10) | 3 (16) | 1 (7) | 0.84 |
| Alcoholism, n (%) | 7 (8) | | 7 (8) | 3 (16) | 2 (14) | 0.84 |
| Diabetes, n (%) | 10 (11) | | 9 (11) | 3 (16) | 2 (14) | 0.91 |
| Immunosuppression, n (%) | 25 (27) | | 22 (26) | 5 (26) | 7 (50) | 0.32 |
| Immunosuppressive therapy, n(%) | 16 (17) | | 14 (17) | 2 (11) | 4 (29) | 0.60 |
| - corticosteroids, n (%) | 12 (13) | | 11 (13) | 0 (0) | 3 (21) | 0.82 |
| - other, n (%) | 3 (3) | | 3 (4) | 2 (11) | 1 (7) | 0.49 |
| Cancer/hemopathy, n (%) | 5 (5) | | 3 (4) | 1 (5) | 2 (14) | 0.43 |
| Other immunosuppressive conditions, n (%) | 6 (7) | | 6 (7) | 1 (5) | 1 (7) | 0.99 |
| ≥1 risk factor | 69 (75) | | 69 (75) | 15 (79) | 11 (79) | 0.72 |
| **Inclusion data** |  | |  |  |  |  |
| ICU admission, n (%) | 60 (65) | | 56 (67) | 19 (100) | 6 (43) | **0.0041** |
| SOFA score, median [IQR] | 3 [1-7] | | 3 [1-6] | 5 [3-9] | 2 [0-6] | **0.05** |
| D0 MV, n (%) | 36 (39) | | 32 (38) | 13 (68) | 6 (43) | 0.92 |
| **D0 laboratory parameters** |  | |  |  |  |  |
| White blood cells (G/L), median [IQR] | 12.7 [8.9-15.8] | | 12.7 [8.9-15.6] | 11.5 [8.9-20] | 10.9 [8.7-14.8] | 0.91 |
| Polynuclear neutrophils (G/L), median [IQR] | 10.8 [7.9-14.6] | | 11.4 [7.9-15] | 9 [6.4-19] | 9.1 [7.5-13] | 0.79 |
| Lymphocytes (G/L), median [IQR] | 0.80 [0.48-1.1] | | 0.82 [0.5-1.1] | 0.78 [0.4-1.2] | 1.1 [0.7-1.5] | 0.51 |
| CRP (mg/L), median [IQR] | 310 [197-421] | | 310 [197-415] | 304 [202-378] | 287 [216-450] | 1 |
| Creatininemia (µmol/L), median [IQR] | 87.5 [71.5-160.3] | | 88.0 [70-160.3] | 103 [73-198] | 80 [74-150] | 0.83 |
| Pulmonary *Legionella* DNA load GU/reaction), median [IQR] | 48.5 [0.9-731.2] | | 55.7 [1.3-662.9] | 75.2 [21.4-248.7] | 82.5 [0.2-610.4] | 0.96 |
| **Clinical data and intensive care during ICU stay** |  | |  |  |  |  |
| Blood pressure (mmHg), median [IQR] | 100 [87-110] | | 100 [87-109.3] | 103 [87-109] | 107 [97-114] | 0.45 |
| Temperature (T°C), median [IQR] | 39 [38.5-40] | | 39 [38.5-40] | 40 [39-40] | 40 [39-40] | 0.89 |
| Vasopressor, n (%) | 20 (22) | | 17 (20) | 6 (32) | 4 (29) | 0.67 |
| Hemofiltration, n (%) | 8 (9) | | 7 (8) | 4 (21) | 1 (7) | 0.36 |
| Corticosteroids (sepsis management), n (%) | 8 (9) | | 8 (10) | 0 (0) | 0 (0) | 0.35 |
| **Evolution** |  | |  |  |  |  |
| Septic shock, n (%) | 21 (23) | | 18 (21) | 6 (32) | 3 (14) | 0.82 |
| D8 MV, n (%) | 23 (25) | | 20 (24) | 11 (58) | 4 (29) | **0.022** |
| Hospitalization duration (d), median [IQR] | 10 [6-26] | | 10 [6-25.8] | 11 [8-31] | 8.5 [6.8-16] | 0.48 |
| ICU duration (d), median [IQR] | 10 [5-16]* | | 9.5 [5-15.3]** | 11 [5-30] | 11 [7.3-17] *** | 0.24 |
| D28 mortality, n (%) | 4 (4) | | 4 (5) | 1 (5) | 0 (0) | 0.87 |

COPD = Chronic Obstructive Pulmonary Disease. Alcoholism corresponds to an alcohol consumption >3 glasses per day for men and >2 glasses per day for women for at least one year. Other immunosuppressive therapies include anti-TNFα therapy (Etanercept, Infliximab, Certolizumab, Adalimumab) and anti-inflammatory biotherapy (Methotrexate). Other immunosuppressive conditions included rheumatoid polyarthritis, solid organ transplantation, and psoriatic arthritis.

IQR: Interquartile range; ICU: Intensive Care Unit; LD: Legionnaires’ disease; SOFA: Sequential Organ Failure Assessment; MV: mechanical ventilation; CRP: C-Reactive Protein; GU: Genome Unit.

* n=58

** n=56

*** n=6


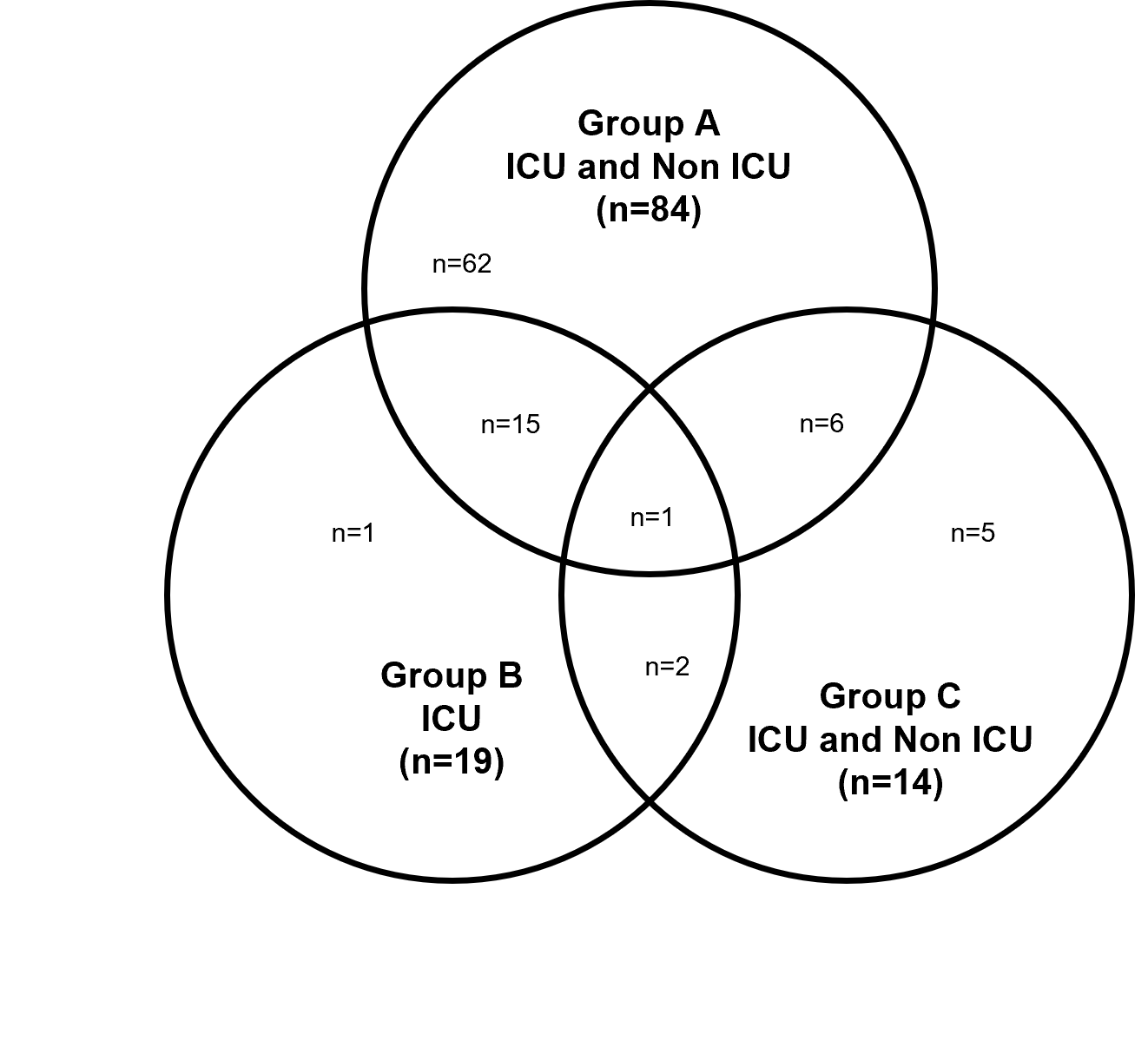


**Figure S1. Group A, B, and C composition.** ICU= Intensive Care Unit

**
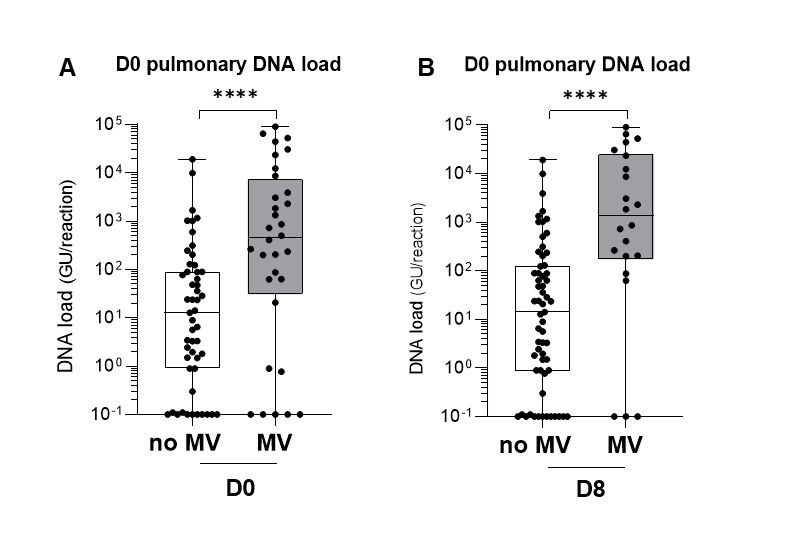
**

**Figure S2. Pulmonary *Legionella* DNA load according to mechanical ventilation (MV) status.** (A) Pulmonary DNA load (GU/reaction) according to D0 MV status (D0 no-MV, n=51, D0 MV, n=32). (B) Pulmonary DNA load (GU/reaction) according to D8 MV status (D8 no-MV, n=61, D8 MV, n=22).

Data are represented as boxplots illustrating the median, interquartile range, and range. Mann-Whitney comparison tests: ****p<0.0001. For graphical representation, values under the quantification limit (≤0.1 copy/reaction) were all figured as equal to 0.1.


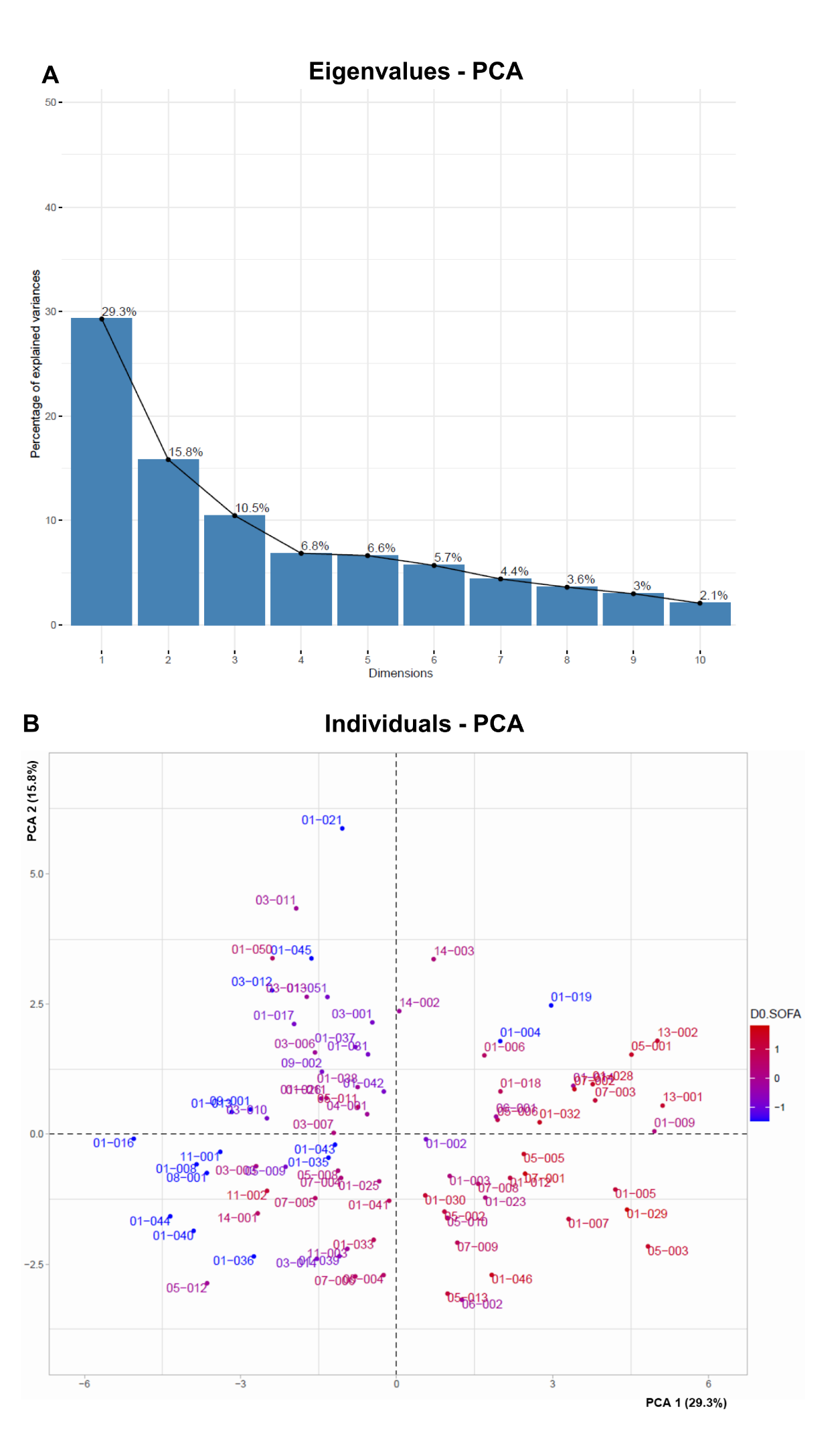


**Figure S3. Eigenvalues and individual values of the PCA.** (A) Percentages of the explained variance or eigenvalues for each axis or dimension of the PCA (B) Projection of the group A individuals (n=84) on the 2 main axes, the D0 SOFA score of individuals to the 2 main axes being represented by the blue to red color gradient


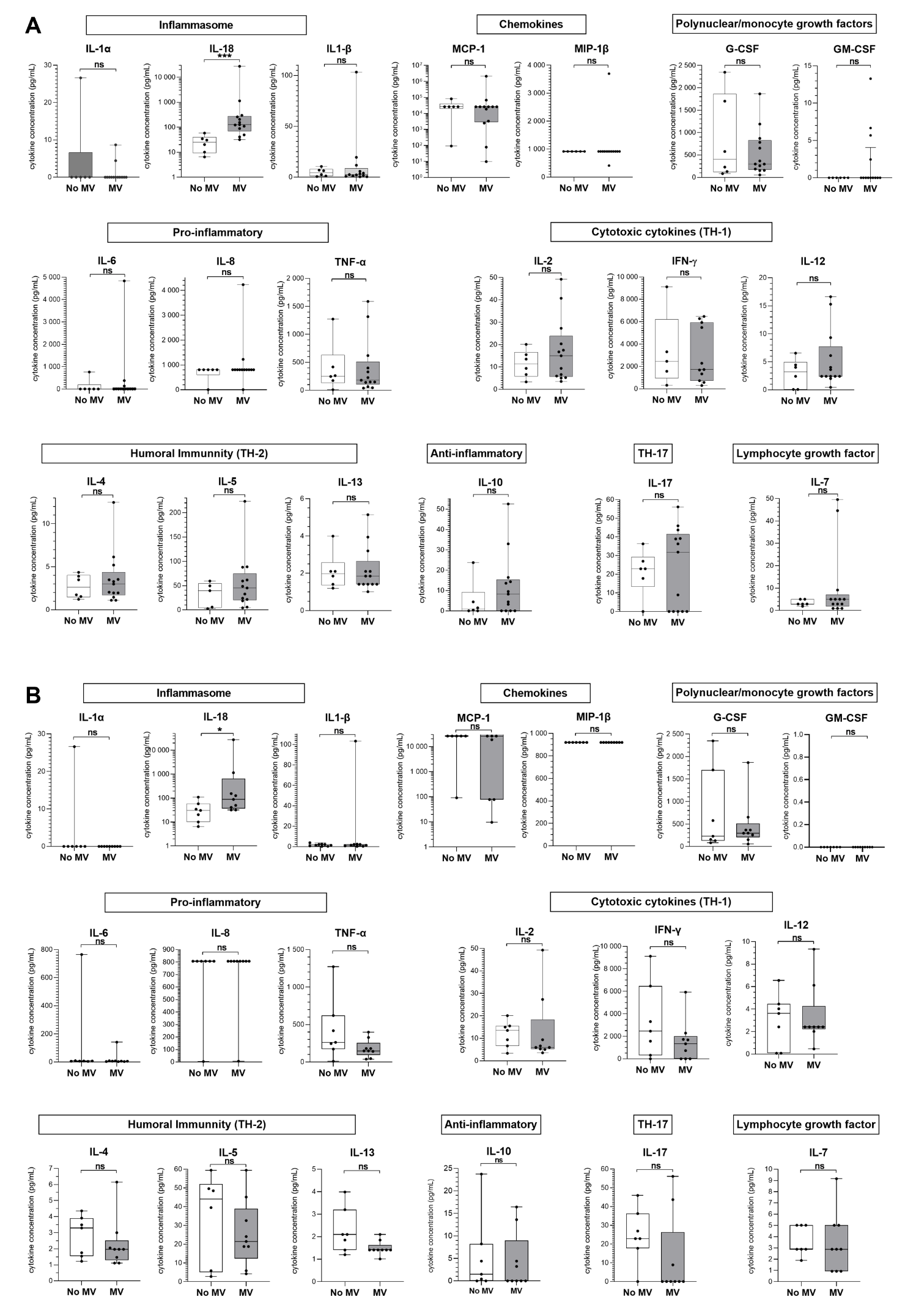


**Figure S4. Concentration of 19 cytokines after conA stimulation according to mechanical ventilation (MV) status in group B patients.** (A) Cytokine expression in D0 no-MV (n=6) vs D0 MV patients (n=13). (B) Cytokine expression in D8 no-MV (n=8) vs D8 MV (n=11) patients. Data are represented as boxplots illustrating the individual values, median, interquartile range, and range; Mann-Whitney comparison tests: ns: non-significant, * p<0.05, *** p<0.001.
